# Effectiveness and safety of electronic cigarettes among sole and dual user vapers in Kuantan and Pekan, Malaysia: a six-month observational study

**DOI:** 10.1186/s12889-018-5951-2

**Published:** 2018-08-20

**Authors:** Mohamad Haniki Nik Mohamed, Azizur Rahman, Shazia Jamshed, Syed Mahmood

**Affiliations:** 1 Department of Pharmacy Practice, Kulliyyah of Pharmacy, International Islamic University of Malaysia (IIUM), Jalan Sultan Ahmed shah, 25200 Kuantan campus, Pahang Malaysia; 2 Department of Clinical Pharmacy, Faculty of Pharmaceutical Sciences, UCSI University, 26000 Cheras, Kuala Lumpur, Malaysia; 3 Department of Pharmaceutical Engineering, Faculty of Engineering Technology, University Malaysia Pahang, 26300 Gambang, Pahang, Malaysia

**Keywords:** Electronic cigarette, Safety, Effectiveness, Conventional cigarette, Carbon monoxide

## Abstract

**Background:**

Current studies on electronic cigarettes (ECs) have assessed the smoking cessation effectiveness and safety of EC among sole EC users. However, in Malaysia and elsewhere, most EC users also smoke conventional cigarettes (CCs). We aimed to investigate nicotine cessation for both ECs and CCs. Additionally, safety issues among sole EC and dual (EC and CC) users over a six-month period were reported.

**Methods:**

We observed 218 sole Malaysian EC and dual users over 6 months from June 2015 to November 2015. Both groups underwent exhaled breath carbon monoxide and saliva cotinine analyses to verify their nicotine cessation from both EC and CC use. Adverse events and withdrawal symptoms were assessed based on self-reports.

**Results:**

Only 3.3% of observed users quit both ECs and CCs, whereas 20.5% quit smoking CCs. Quitting ECs and CCs was significantly higher among sole EC users (5 vs 2, respectively; OR: 5.62; *P* = 0.036) than it was among dual users, a result that was similar for CCs smoking (29 vs. 15; OR: 6.33; *P* ≤ 0.001). No severe health issues were reported over the entire study period.

**Conclusion:**

The rates of quitting CCs and ECs were higher in sole EC users than those in dual users. No serious health effects were reported over 6 months in either group. ECs may serve as a smoking cessation aid in Malaysia, but appropriate regulations are necessary to encourage sole EC use to ensure product quality. Large randomised clinical trials (RCTs) with a longer follow-up are required to better measure the effectiveness and safety of ECs use alone and in combination with CCs.

## Background

Consumer interest and the use of electronic cigarettes (ECs) as a substitute for smoking have intensified exponentially in the past few years. The popularity of ECs is increasing both globally and in Malaysia [1, 2]. ECs are battery-powered devices that enable the vaporisation of a solution containing propylene glycol, glycerol, and some flavouring agents, with or without nicotine. There is significant progress in the technology of EC devices. Currently, ECs are categorised generation wise. First-generation ECs are identical to conventional cigarettes (CCs) and are called cigalikes. Second-generation ECs are comparable to fountain pens and are named vape pens, and third-generation ECs are termed advanced personal vaporisers (APVs) or mods [2, 3]. In Malaysia, most vapers use third-generation ECs [4]. ECs do not contain tobacco leaves. Therefore, the vapours produced from them are free from carbon monoxide and tars.

Many individuals who use ECs (also referred to as vapers) believe that using ECs is safer than smoking CCs. Therefore, vapers consider that ECs can be used as a simply harmless approach to quit smoking [5–7]. Currently, high-quality randomised clinical trials are still limited to determine the exact role of ECs in smoking cessation [8–10]. The Cochrane database and some meta-analyses have revealed that, under the GRADE system, the overall quality of evidence is low due to the small number of trials [11–14]. Presently, the implementation of EC randomised clinical trials (RCTs) is challenging due to the paucity of safety data and difficulty in obtaining ethical approval. Additionally, EC technology is continuously upgrading, and many new EC devices are entering the market. Therefore, it may be possible that the EC product used in the RCTs may no longer be available by the time the study results are released. By contrast, it may be possible that the new EC devices may offer better assistance in managing adverse effects and smoking-related withdrawal symptoms than do the products used in RCTs [15]. Moreover, the RCTs are generally performed under controlled environments that may not be generalisable to real-world situations. Consequently, in realistic situations, elements such as appeal, accessibility, price and vaping community norms are essential dictators of EC use. Until RCTs are available, prospective observational studies are pertinent to gathering information about EC effectiveness and its hidden safety issues in human beings. Presently, regulatory authorities are not in favour of EC use due to the lack of extended-period studies [16]. Furthermore, the regulatory authorities fear that EC use may reduce a smoker’s motivation to quit smoking and renormalise smoking in ex-smokers. Additionally, some previous studies have shown that ECs may increase the risk of nicotine addiction in adolescents and women due to the appeal of various available flavours [17–19]. Presently, in Malaysia, ECs are not banned per se but are regulated with only nicotine-free e-liquids permitted to be sold by vendors. However, as per the Poisons Act 1952, nicotine in e-liquids must be sold by licensed personnel, including pharmacists and physicians [20]. The recent Malaysian National E-cig Survey (NECS, 2016) reported that the prevalence of EC users among Malaysians aged 18 years and above was 3.2%. The incidence of urban users was 3.3% and that of rural users was 2.9%. However, the incidence of dual users was 2.3%, comprising 2.5% urban and 1.8% rural [4].

Currently, studies are required to examine the long-term safety and effectiveness of EC in a naturalistic setting among sole EC and dual users (EC and CC use). Because both sole EC and dual users represent the real-world population of the vaper community, most vapers use ECs with CCs [4]. However, studies are required to reveal whether the combined use of ECs along with CCs is more likely to lead to smoking termination or upsurge the nicotine addiction. Additionally, studies are required to explore the dual use of ECs that reinforces both behaviours among vapers. In our previously published study, a higher number of sole EC users quit CCs than that of dual users. However, the study did not report a single nicotine-free participant in either group [21]. Tobacco control authorities have recommended that smoking cessation studies should not only aim to promote CC quitting but abstinence from nicotine use should be the crucial goal [15]. Thus, the primary aim of this study was to evaluate complete cessation from both ECs and CCs. The safety issues among the sole EC and dual users over a six-month period were also measured. The data were collected from sole EC and dual users in two Malaysian districts, Kuantan and Pekan, between March 2015 and November 2015.

## Methods

### Study design

A one-month observational study among Malaysian EC users was previously conducted [21]. The current study extended this earlier work and explored the long-term safety and effectiveness of ECs in the same cohort for up to 6 months. Therefore, this 24-week observational study consisted of three visits—at baseline and two follow-up sessions at weeks 4 and 24, respectively.

### Inclusion and exclusion criteria

The inclusion criteria were existing sole EC and dual users using ECs for at least 1 month before enrolment in the study, age > 18–65 years with good self-reported health, and agreement to sign the consent form and follow the study procedures. Exclusion criteria included the use of any smoking cessation medicines—e.g., nicotine replacement therapy (NRT) or varenicline—currently or within the last year. Subjects who were dependent on any illegal drugs were also excluded.

### Study questionnaire

A pre-validated interview-administered English questionnaire was used for the data collection. The survey was developed and piloted on Malaysian populations as described previously [21]. The survey consisted of questions regarding the demographic characteristics of participants, queries, and measurements related to the evaluation of the effectiveness and safety of ECs. In this study, two improvised validated scales were applied the EC modified Fagerstrom Test for Nicotine Dependence (EC-MFTND) and EC modified Glover-Nilsson Vaping Behavioural Questionnaire (EC-MGNVBQ). The newly developed scales assessed and identified the physical and behavioural dependence to nicotine administered via ECs [21].

### Sample size

To detect a 10% smoking cessation rate with 80% power in the whole study population, a sample of 200 subjects was required [22, 23]. A 10% quit rate chosen in some earlier RCTs on EC revealed comparable abstinence rates among their study populations [8, 9].

### Recruitment of participants and settings

**Fig. 1:**
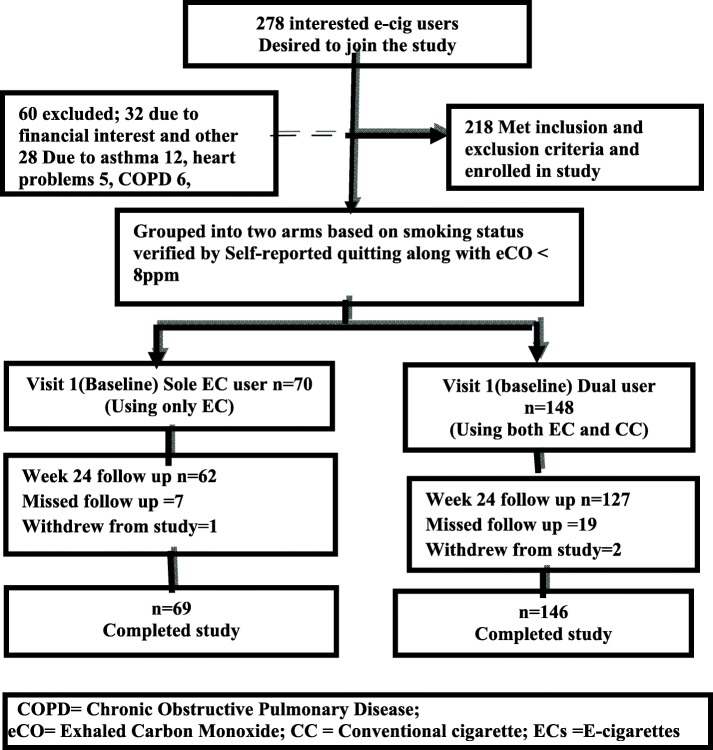
Flow chart displaying recruitment and follow up of e-cigarettes users

The study subjects were enrolled from the semi-urban districts of Kuantan and Pekan, in the state of Pahang, Malaysia. As per the National Health and Morbidity Survey (NHMS) 2015, the smoking prevalence in the state of Pahang was 25.5%, which was higher than the national smoking prevalence rate of 22.8% [24]. These two locations were chosen due to the feasibility of the research sites concerning time, funding constraints and balance regarding the accessibility of EC users. We informed the applicants that there would be no monetary compensation for enrolment in the research, but reimbursement could be provided for food and transport expenses. More than 90% of the subjects could understand and speak English. However, a few subjects used a professional translator who was equally proficient in both languages. The same professional translator was used in all the visits to avoid any prejudice and inconsistencies related to documentation. Figure 1 shows the process involved in the recruitment of subjects.

### Data collection

At baseline, the participants were categorised into two arms—sole EC users and dual users—based on the smoking status. The sole EC users used only ECs and had a carbon monoxide (CO) level ≤ 7 ppm (ppm). However, dual users used both ECs and CCs and had a CO value ≥8 ppm. At the baseline and follow-up appointments, both groups were verified by the exhaled breath CO level. At week 24, in addition to CO testing, the saliva cotinine level was measured to check the nicotine-free status. Users in both groups were interviewed further to collect information regarding the adverse effects, any smoking-related illnesses and withdrawal symptoms that could appear due to EC use. The participants were questioned individually for approximately 25–30 min to maintain confidentiality and to ensure that their answers were not being disclosed to other participants.

### Data analysis

Categorical variables were summarised as frequencies and percentages, whereas continuous variables were calculated as medians because medians are less sensitive to extreme values. Statistical analysis was performed based on the intention to treat (ITT). Chi-squared test applied categorical variables, whereas independent t-tests were used to compare the mean differences between the groups. The Mann-Whitney U test was used to analyse the nonparametric data between the group users. However, the Wilcoxon signed-rank test was applied within the same group. Statistical methods were two-tailed, and a *p*-value less than 0.05 was considered significant. The analyses were performed using the Statistical Package for Social Sciences (IBM®, SPSS® Inc., Chicago, IL) for Windows version 21.

## Results

Two hundred eighteen participants enrolled in the study. One hundred forty-eight (148) were recognised as dual users, while 70 were sole EC users. However, 215 subjects were considered for the intention to treat (ITT) analysis because three subjects withdrew during the study period—two dual users and one sole EC user.

### Baseline characteristic of the participants

No significant difference was found in the demographics of both groups as outlined in Table 1. Almost 95% of the participants in both groups had not attempted any previous therapy to stop smoking. However, less than 5% of participants had used nicotine replacement therapy more than 1 year ago, and the median age of the onset of smoking in both groups was 15 years (*P* = 0.125). The median CCs use pack per year was 7 among dual users compared with 5 in sole EC users. However, this difference was not significant (*P* = 0.114). The median duration of EC use among single EC users was 8 months compared with 4 months for dual users, and the difference was significant (*P* < 0.001). However, at baseline, the comparison between both groups for physical and behavioural dependence to ECs by EC-MFTND and EC-MGNVBQ scales revealed no significant difference. More than 98% of the participants used EC daily, and only 1.4% used them occasionally (*P* = 1.000). More than 95% in both groups reported the use of third-generation EC models (Mods), and the remaining less than 5% reported using second-generation EC vape pens. The difference was insignificant. All participants reported using nicotine EC at concentrations ranging from 6 to 18 mg/ml.

**Table 1:** Baseline characteristics and electronic cigarettes status of the study participants

Characteristics	Dual users *n* = 146	Single user *n* = 69	Total participants (*n* = 215)	*P* value (2-tailed)
Age	23 (19–40)	23 (19–39)	23 (19–40)	0.946*
Sex				
Male	145 (98)	69 (98.6)	214 (98.2)	1.0
Female	3 (2)	1 (1.4)	4 (1.8)	
Marital status				
Married	22 (14.9)	19 (27.1)	41 (18.8)	0.041
Single	126 (85.1)	51 (72.9)	177 (81.2)	
Race				
Malay	127 (85.8)	61 (87.1)	188 (86.2)	0.632
Chinese	19 (12.8)	7 (10)	26 (11.9)	
Indian	2 (1.4)	2 (2.9)	4 (1.8)	
Education				
Primary	–	–	–	
secondary	39 (26.4)	19 (27.1)	58 (26.6)	0.902
Diploma/Degree	109 (73.6)	51 (72.9)	160 (73.4)	
Occupation				
Private	70 (47.3)	38 (54.3)	108 (49.5)	0.535
Government	13 (8.8)	6 (8.6)	19 (8.7)	
Self-employment	15 (10.1)	9 (12.9)	24 (11)	
Students	50 (33.8)	17 (24.3)	67 (30.7)	
Income				
00^♀^	50 (33.8)	17 (24.3)	67 (30.7)	0.468
< 2500 MYR^#^	2 (1.4)	2 (2.9)	4 (1.8)	
≥ 2500–5000 MYR	89 (60.1)	48 (68.6)	137 (62.8)	
> 5000 MYR	7 (4.7)	3 (4.3)	10 (4.6)	
EC-MFTND	3 (1–7)	3 (1–7)	3 (1–7)	0.668
EC-MGNVBQ	14 (3–22)	13 (3–24)	14 (3–24)	0.625
Quit attempts in the past year?	0 (0–1)	0 (0–1)	0 (0–1)	0.585*
Any therapy to quit smoking before EC use				
Never Attempted	142 (95.9)	66 (94.3)	208 (95.4)	0.730
NRT	6 (4.1)	4 (5.7)	10 (4.6)	
Age started smoking	15 (12–27)	16 (12–22)	15 (12–27)	0.125*
Pack/year	7 (0.5–50)	4.75 (0.25–30)	6 (0.25–50)	0.114*
ECs use (months)	4 (2–24)	8 (6–24)	5 (2–24)	< 0.001*
ECs consumption				
Daily	145 (98)	69 (98.6)	214 (98.2)	1.000
Occasionally	3 (2)	1 (1.4)	4 (1.8)	

*Non-parametric data were expressed as medians, and *p* values were calculated using the Mann-Whitney U test; for categorical variables, chi-squared test was applied to calculate the value. *EC-MFTND* EC-Modified Fagerstrom Test for Nicotine Dependence, *EC-MGNVBQ* EC-Modified Glover-Nilsson Vaping Behavioural Questionnaire

### Evaluation of EC effectiveness

**Fig. 2:**
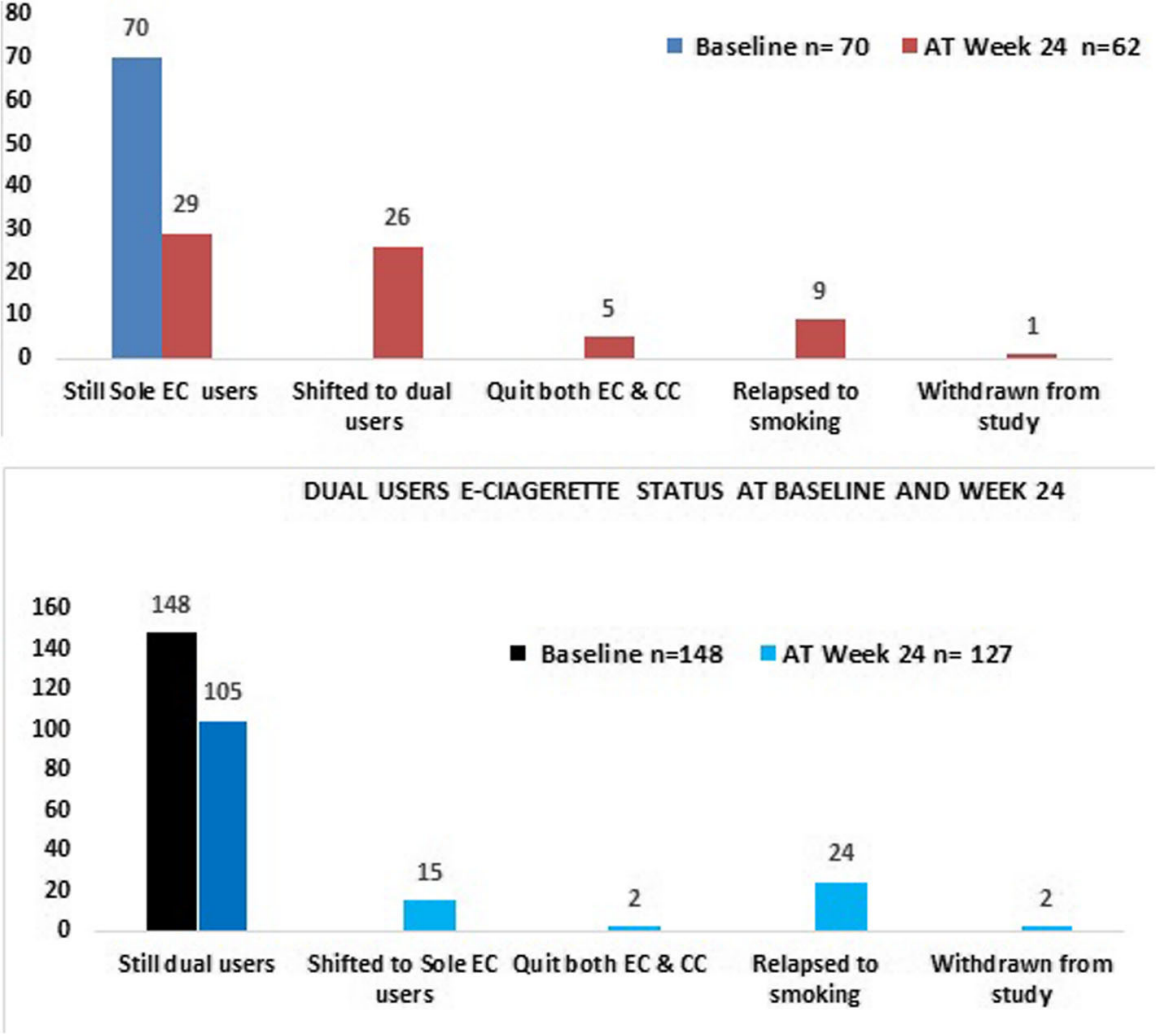
Sole EC users status at baseline & at week 24

Table 2 displays the effectiveness of ECs between the groups. The median intake of CCs in both groups before EC use was 20 CCs per day. Among 148 dual users after the six-month follow-up, 105 (71.91%) remained dual users, 24 (16.43%) relapsed to smoking, 15 (10.3%) shifted to become sole EC users, two (1.4%) reported to quit both ECs and CCs, and 2 withdrew from the study. However, among the 70 sole EC users, 29 (42%) continued to be sole EC users, 9 (13.04%) completely relapsed to smoking, 26 (37.68%) shifted to becoming dual users, 5 (7.2%) totally quit both EC and CC use, and one withdrew from the study (Fig. 2). There was no significant reduction in the CC consumption rate for the dual users at baseline and at week 24 (*P* = 0.087). However, among the sole EC users, who relapsed to smoking, their CC consumption per day remained lower than that of dual users (*P* < 0.001).

**Table 2:** Evaluation of electronic cigarettes effectiveness

Conventional cigarette (CCs) Consumption and exhaled carbon monoxide (CO) Level at baseline				
Characteristics	Dual users *n* = 146	Sole EC users *n* = 69	Statistic	*P* value (2-tailed)
CC/day before using EC	20 (10–60)	20 (5–40)	U = 4412.000	0.055
CC/day at baseline visit 1	5 (1–30)	–	Z = −9.281	< 0.001
Exhale Carbon Monoxide (CO)	8 (8–40)	3 (1–6)	U = 1577.500	< 0.001
Conventional cigarettes consumption and CO level at week 24				
Characteristics	Dual users *n* = 127	Sole EC users *n* = 62	Statistic	*P* value (2-tailed)
CC/day at baseline vs week 24	5 (1–30)	–	Z = −1.710	0.087
CC/day at week 24	6 (0–40)	0 (0–20)	U = 1842.00	< 0.001
CO at week 24	8 (2–20)	3 (2–11)	U = 1650.50	< 0.001
Evaluation of Electronic cigarettes effectiveness by the seven-day prevalence abstinence rate at week 24 (Intention To Treat (ITT) analysis)				
At Week 24	% (n)		OR (95% CI)	*P* value (2-tailed)
Quit only CCs				
Both users	20.5% (44 of 215)			
Sole EC users	42% (29 of 69)		6.33 (4.09-	< 0.001
Dual user	10.3% (15 of 146)		0.646)	
Quit both ECs and CCs				
Both users	3.3% (7 of 215)		5.62 (5.29-	0.036
Sole EC users	7.2% (5 of 69)		0.940)	
Dual users	1.4% (2 of 146)			

Odd ratio (OR), 95% confidence interval (95% CI)

*CCs* conventional cigarettes, *CO* carbon monoxide

Non-parametric data were expressed as medians, and *p* values were calculated by the Mann-Whitney U test between both groups

The CO median levels for dual users during the entire study period were significantly higher than that of sole EC users (P < 0.001). The median CO levels of only EC users at baseline were lower than those of dual users—i.e., 3 vs. 8 ppm—and it was statistically significant. According to PiCO+ smokerlyzer® manual issue 2, 2012 (Bedfont Scientific Limited Maidstone, UK), a non-smoker value is between 0 and 6 ppm [25]. However, in the current study, a cut-off value of 7 ppm was applied because all sole EC users showed exhaled CO values between 3 and 7 ppm. Additionally, a 7-ppm cut off value was specified to differentiate EC users from CC users in previous studies [9, 26]. Again, at week 24, the sole EC users had lower levels of CO than did the dual users—i.e., 3 vs. 8 ppm, respectively—and the difference was significant (*P* < 0.05). The subjects were considered completely nicotine free when they quit both EC and CC use and showed CO levels ≤7 ppm and a zero level on the saliva NicAlert® scale [27]. A participant with a CO level ≤ 7 ppm but a saliva cotinine level ≥ 1 was considered only a CC quitter. Because quitters of both ECs and CCs should show a zero value on NicAlert® scale— i.e., ≤ 10 ng/ml—the six-month follow-up revealed that 20.5% (44 of 215, ITT analysis) of users in both groups abstained from CCs. However, only 3.3% (7 of 215, ITT) quit both ECs and CCs use. Sole EC users showed a higher abstinence rate for CCs (29 vs. 15; OR: 6.33; *P* ≤ 0.001) and for combined ECs and CCs (5 vs. 2; OR: 5.62; *P* < 0.036) than that for dual users, and the difference was statistically significant. The overall smoking status of users in both groups at the baseline and week 24 is displayed in Fig. 2.

### Measures of EC safety

#### Measure of EC adverse effects

Dry mouth was reported by more than 50% of all participants and was reported to be the strongest adverse effect in our study. The other statistically non-significant adverse effects reported by users in both groups during the six-month period are shown in Table 3. The significant adverse effects reported by the dual users were cough and breathing problems at baseline and week 24 (*P* < 0.05) compared with the sole EC users. Interestingly, some cases of vomiting and fever were reported in sole EC users during the baseline visits but not at week 24. Two instances of gingivitis in sole EC users were also documented. Overall, dual users experienced more adverse effects than did sole EC users during the entire study period. Additionally, the safety of EC was observed for diseases related to smoking between users in both groups. Illnesses such as chronic obstructive pulmonary disease (COPD), asthma, heart diseases, stroke, hypertension, diabetes, thyroid disease, cataracts and other serious conditions were also observed. One case of COPD was documented in the dual user group, whereas no such cases were reported in sole EC users during the entire study period.

**Table 3:** Adverse events experienced by both groups at baseline and at week 24. Scale: 4 = severe, 3 = Moderate, 2 = Mild, 1 = slight, 0 = absent; ^a^Fisher’s exact test

Adverse events	Groups	At baseline			At week 24		
		Total n (%)	Mean	P (2-tailed)	Total n (%)	Mean	P (2-tailed)
Dry Mouth	Dual user Sole EC user	82 (55.4) 30 (42.9)	1.229 0.900	0.062	21 (16.5) 09 (14.5)	.2205 .2097	0.904
Sore throat	Dual user Sole EC user	32 (21.6) 9 (12.9)	.3986 .2714	0.262	13 (10.2) 8 (12.9)	.1102 .1290	0.720
Cough	Dual user Sole EC user	34 (23) 8 (11.4)	0.5473 0.1571	< 0.001	31 (24.4) 7 (11.3)	.3858 .1452	0.009
Anxiety	Dual user Sole EC user	0 (0) 1 (1.4)	.0000 .0286	0.321	–	–	–
Stomach disturbances	Dual user Sole EC user	2 (1.4) 0 (0)	.0270 .0000	0.158	–	–	–
Nausea	Dual user Sole EC user	–	–	–	6 (4.7) 2 (3.2)	.0551 .0323	0.535
Vomiting	Dual user Sole EC user	0 (0) 5 (7.1)	0.000 0.1571	0.033	3 (2.4) 2 (3.2)	.0394 .0484	0.830
Headache	Dual user Sole EC user	14 (9.5) 6 (9.2)	.1486 .1286	0.771	5 (3.9) 5 (8.1)	.0551 .0968	0.388
Breathing Problem	Dual user Sole EC user	27 (18.8) 2 (2.8)	.3243 0429	< 0.001	24 (18.9) 4 (6.5)	.2598 .0806	0.008
Other (fever, gums bleeding	Dual user Sole EC user	0 (0) 3 (4.3)^a^	.0000 .0571	0.103	1 (0.8) 2 (3.2)^b^	.1102 .0806	0.775

^a^Fever cases; ^b^Gum bleeding

Independent t-test was applied to compare the mean differences between the groups

#### Measure of EC withdrawal symptoms

The most commonly observed withdrawal symptom was the craving for smoking. The urge to smoke was reported more often by dual users than by sole EC users at the baseline visit (*P* < 0.001) but not at week 24 (*P* = 0.093). By contrast, a high appetite was reported more in the sole EC users than that in the dual users at the baseline visit but not at the 6-month follow-up (baseline *P* = 0.038; week 24 *P* = 0.773). Other non-significant withdrawal symptoms documented in both groups included depression, difficulty concentrating, bad temper, sleeplessness, sleepiness, frustration, anger and awakening at night (for all *P* > 0.05). A long interval in the disappearance of withdrawal symptoms was documented in the dual users compared with that in sole EC users during the entire study period.

## Discussion

The current study is the first long-term observational study to examine the effectiveness of ECs in assisting with smoking cessation in Malaysian populations. A six-month follow-up study revealed that nearly a quarter of the total study participants abstained from CCs smoking. However, the smoking quit rate was noticeably higher among sole EC users than that among dual users. Previous studies have suggested that ECs helped many tobacco users to quit smoking, or at least its use lowered the consumption of CCs [5, 6]. The smoking cessation rate recorded in the current research was higher than that in previous clinical trials [9, 10]. The cause might be that, in this type of observational study, the participants who perceived positive effects from ECs were more highly motivated to enrol in the study than those who encountered unwanted effects from vaping.

Therefore, the results should be interpreted with caution when comparing with other population studies. The varying results might also be due to the utilisation of diverse ECs generations models and puffing topography among the study participants. Earlier studies have reported that newer ECs models and different puffing topographies among the participants affect vaper fulfilment towards craving for CCs smoking [28, 29]. Nevertheless, the effectiveness of these devices in assisting in smoking cessation is necessary along with ECs topography studies among the diverse ECs user populations. Additionally, advance research is necessary to ascertain the effectiveness of ECs with or without nicotine among current vapers, and regulatory laws must be applied regarding the sale of nicotine ECs to avoid misuse among non-smokers and adolescents.

The current study revealed 20% CCs abstinence but reported a low rate (3.3%) of combined ECs and CCs cessation among both groups after a six-month period. It is pertinent that using ECs even for longer period did not lower the rates of nicotine addiction, suggesting that most users simply used ECs as an alternative device for the intake of nicotine and further supported by the use of nicotine ECs by nearly all the study participants. Therefore, it is evident that nicotine plays a vital role in the success of ECs as a smoking aid, a finding that was also demonstrated in previous studies [30–32]. The possibility cannot be ruled out that EC use required more time to be free from nicotine addiction than that of therapies already approved by the Food and Drug Administration (FDA) for tobacco cessation. Currently, it is debatable for the researchers regarding the status of nicotine ECs as a smoking cessation aid compared with other conventional therapies [14]. The preceding mentioned issues point towards a confounding appearance of ECs in smoking cessation control. It is possible that vaping may uphold nicotine addiction, could renormalise smoking habits and may disturb a smoker’s passion for quitting smoking. The study showed no difference in the intake of CCs at the baseline and at the final visit among dual users; therefore, this labels that the addition of ECs to current smoking did not encourage a reduction of CCs smoking. Previous studies also reported that sole regular use of ECs leads to a significantly higher quit rate than that for dual use [33, 34].

The strongest adverse effect experienced in both the groups was dry mouth, followed by sore throat, anxiety, nausea and stomach disturbances. However, the highest noticeable withdrawal symptom was craving for smoking, reported more by dual users than it was by sole EC users. The side effects reported here are consistent with those found in other studies [5, 6, 8]. Moreover, one incident of COPD was documented in a dual user. No such cases were reported in sole EC users during the entire study period. Regarding smoking history data, the subject diagnosed with COPD was a heavy smoker in the past, and his CC pack/year was 24. Therefore, the incident of COPD was more likely related to his extensive smoking history. However, currently, there is confusion among consumers, health professionals and researchers about the safety of ECs. Existing available studies reveal the level of risk from ECs is lower than that of CCs. However, with the available data, it is not possible to compute extended health hazards of ECs with those of CCs. Therefore, long-term studies are urgently required to explore the harmful effects of compounds that are released from EC vapours [35–38]. In fact, more research is required to examine whether EC is as safe as other conventional smoking cessation therapies.

### Limitations of the study

The limitations of the current research were mostly related to the sample size and demographics of the study population. Most of the participants were middle-aged Malay males, with fewer Chinese and Indians from the Kuantan and Pekan districts of Malaysia. All the participants were otherwise healthy smokers. Thus, the findings may not be generalisable to other populations of EC users, including female smokers with health issues and those of different races. Moreover, in this study, the subjects were recruited based on their motivation to quit smoking, which also limits the generalisation of our results. However, the current observational study is of significant relevance depicting the real understanding, benefits, and undesirable effects among sole ECs and dual users of Malaysian vapers.

The study also reflected that the study participants were persistent smokers who were unwilling to quit smoking by the currently approved FDA therapy. Therefore, for such subjects, pharmacotherapy may not be the treatment of first choice. In the current study, during follow-up visits, we collected the data from more than 85% of subjects and missed nearly 15% of those at week 24. Therefore, it may be possible that the missing items were due to quitting both ECs and CCs, which would alter the reported results. However, this finding can be anticipated in any long-term study. The severity of the adverse events, withdrawal symptoms and smoking-related diseases reported during all the visits were based on the subjects own experience and were not being independently evaluated by an investigator. No physical examinations were conducted to judge the severity of these effects and diseases.

## Conclusion

The current study revealed a good CC cessation rate among sole EC and dual users but reported low abstinence for combined ECs and CCs use. There was no significant difference in CCs consumption for dual users at baseline and after the six-month follow-up period. This indicated that using ECs along with CCs does not aid CCs reduction. The major adverse effects and withdrawal symptoms observed among dual users were coughing, breathing problems and craving for smoking. Overall, sole EC users perceived fewer adverse effects and withdrawal symptoms than did dual users. Therefore, ECs may be used as an additional supportive smoking cessation aid in Malaysia, but appropriate procedures are necessary to encourage sole EC use and product quality. Large RCTs with a longer follow-up are required to better understand the effectiveness and safety of these novel products in combination with CCs among diverse populations.

## Abbreviations

CC: Conventional cigarette
CO: Carbon monoxide
DU: Dual users
EC: Electronic cigarette
IIUM: International Islamic University of Malaysia
IREC: International Islamic University of Malaysia Research Ethics Committee
ITT: Intention To Treat
NMRR: National Medical Research Registration
NRT: Nicotine Replacement Therapy
PPM: Parts Per Million
RCTs: Randomised Clinical Trials
SPSS: Statistical Package for Social Sciences

## References

[1] Gravely S, Fong GT, Cummings KM, Yan M, Quah AC, Borland R, Yong HH, Hitchman SC, McNeill A, Hammond D, Thrasher JF (2014). Awareness, trial, and current use of electronic cigarettes in 10 countries: findings from the ITC project. Int J Environ Res Public Health.

[2] Palipudi KM, Mbulo L, Morton J, Bunnell R, Blutcher-Nelson G, Kosen S, Antoniadou E (2015). Awareness and current use of electronic cigarettes in Indonesia, Malaysia, Qatar, and Greece: findings from 2011–2013, Global Adult Tobacco Surveys. Nicotine Tob Res.

[3] Grana RA, Ling PM, Benowitz N, Glantz S (2014). Electronic cigarettes. Circulation.

[4] National E-cigarette survey (NECS) 2016: Prevalence, pattern and perception regarding E- cigarette and vape use among Malaysian adults. Institute of public health and IIUM. 2017. ISBN 978-983-2387-33-6.

[5] Etter JF, Bullen C (2014). Longitudinal study of electronic cigarette users. Addict Behav.

[6] Farsalinos KE, Romagna G, Tsiapras D, Kyrzopoulos S, Voudris V (2014). Characteristics, perceived side effects and benefits of electronic cigarette use: a worldwide survey of more than 19,000 consumers. Int J Environ Res Publ Health.

[7] Polosa R, Morjaria JB, Caponnetto P, Campagna D, Russo C, Alamo A, Amaradio M, Fisichella A (2014). Effectiveness and tolerability of electronic cigarette in real-life: a 24-month prospective observational study. Intern Emerg Med.

[8] Adriaens K, Van Gucht D, Declerck P, Baeyens F (2014). Effectiveness of the electronic cigarette. An eight-week flemish study with six-month follow-up on smoking reduction, craving and experienced benefits and complaints. Int J Environ Res Publ Health.

[9] Bullen C, Howe C, Laugesen M, McRobbie H, Parag V, Williman J, Walker N (2013). Electronic cigarettes for smoking cessation: a randomised controlled trial. Lancet.

[10] Caponnetto P, Campagna D, Cibella F, Morjaria JB, Caruso M, Russo C, Polosa R (2013). EffiCiency and safety of an eLectronic cigAreTte (ECLAT) as tobacco cigarettes substitute a prospective 12-month randomized control design study. PLoS One.

[11] Hartmann-Boyce J, McRobbie H, Bullen C, Begh R, Stead LF, Hajek P. Electronic cigarettes for smoking cessation. Cochrane Database Syst Rev. 2016; Issue 9. Art. No: CD010216. DOI: 10.1002/14651858.CD010216.pub3. 10.1002/14651858.CD010216.pub3 PMC6457845 27622384

[12] Khoudigian S, Devji T, Lytvyn L, Campbell K, Hopkins R, O’Reilly D (2016). The efficacy and short-term effects of electronic cigarettes as a method for smoking cessation: a systematic review and a meta-analysis. Int J Public Health.

[13] Kalkhoran S, Glantz SA (2016). E-cigarettes and smoking cessation in real-world and clinical settings: a systematic review and meta-analysis. Lancet Respir Med.

[14] National Academies of Sciences, Engineering, and Medicine (2018). Public health consequences of E-cigarettes.

[15] McNeill A, Brose LS, Calder R, Hitchman SC, Hajek P, McRobbie H. ECarettes an evidence update. A report commissioned by *Public Health England*. 2015 < www. gov. UK/government/uploads/system/uploads/attachment data/file/454516/ECarettes an evidence update A report commissioned by Public Health England. pdf> (Accessed August 22, 2015).

[16] Künzli N (2014). To e-smoke or not to e-smoke: is that a question?. Int J Public Health.

[17] Alawsi F, Nour R, Prabhu S (2015). Are ECarettes a gateway to smoking or a pathway to quitting?. Br Dent J.

[18] Czoli CD, Hammond D, White CM (2014). Electronic cigarettes in Canada: prevalence of use and perceptions among youth and young adults. Can J Public Health.

[19] McMillen RC, Gottlieb MA, Shaefer RM, Winickoff JP, Klein JD (2014). Trends in electronic cigarette use among US adults: use is increasing in both smokers and non-smokers. Nicotine Tob Res..

[20] Control of drugs and cosmetics regulations. 1952. Retrieved from https://www.pharmacy.gov.my/v2/sites/default/files/document-upload/sales-drug-act-1952-act-368.pdf.

[21] Rahman A, Nik Mohamed MH, Jamshed S (2016). Evaluating effectiveness and safety toward electronic cigarette among Malaysian vapers: One-month observational study. Arch of Phar Practice.

[22] Chow SC, Wang H, Shao J (2007). Sample Size Calculations in Clinical Research.

[23] Machin D, Campbell MJ, Tan SB, Tan SH (2011). Sample Size Tables for Clinical Studies.

[24] Institute for Public Health (IPH) (2015). National Health and Morbidity Survey 2015 (NHMS). Vol. II: Non-Communicable Diseases, Risk Factors & Other Health Problems.

[25] PiCO^+^™ Smokerlyzer® operating manual. Retrieved from https://www.bedfont.com/file.php?f=ZmlsZSMjNzE0.

[26] Jarvis MJ, Tunstall-Pedoe H, Feyerabend C, Vesey C, Saloojee Y (1987). Comparison of tests used to distinguish smokers from non-smokers. Am J Public Health.

[27] Cooke F, Bullen C, Whittaker R, McRobbie H, Chen MH, Walker N (2008). Diagnostic accuracy of NicAlert cotinine test strips in saliva for verifying smoking status. Nicotine Tob Res.

[28] Behar RZ, Talbot P (2015). Puffing topography and nicotine intake of electronic cigarette users. PLoS One.

[29] Farsalinos KE, Spyrou A, Tsimopoulou K, Stefopoulos C, Romagna G, Voudris V. Nicotine absorption from electronic cigarette use: comparison between first and new-generation devices. Sci Rep. 2014;4:4133. 10.1038/srep04133. 10.1038/srep04133 PMC3935206 24569565

[30] Benowitz NL. Neurobiology of nicotine addiction: implications for smoking cessation treatment. Am J Med. 2008;121(4):S3–10. 10.1016/j.amjmed.2008.01.015 18342164

[31] D’souza MS, Markou A (2011). Neuronal mechanisms underlying development of nicotine dependence: implications for novel smoking-cessation treatments. Addict Sci Clin Pract.

[32] Markou A (2008). Neurobiology of nicotine dependence. Philos Trans R Soc Lond B Biol Sci.

[33] Brown J, Beard E, Kotz D, Michie S, West R (2014). Real-world effectiveness of e-cigarettes when used to aid smoking cessation: a cross-sectional population study. Addiction.

[34] Manzoli L, Flacco ME, Fiore M, La Vecchia C, Marzuillo C, Gualano MR, Liguori G, Cicolini G, Capasso L, D'Amario C, Boccia S (2015). Electronic cigarettes efficacy and safety at 12 months: cohort study. PLoS One.

[35] Allen JG, Flanigan SS, LeBlanc M, Vallarino J, MacNaughton P, Stewart JH, Christiani DC (2016). Diacetyl, 2, 3-Pentanedione, and acetoin in a sample of 51 products, including fruit, candy and cocktail Flavoured ECarettes. Environ Health Perspect.

[36] Bekki K, Uchiyama S, Ohta K, Inaba Y, Nakagome H, Kunugita N (2014). Carbonyl compounds generated from electronic cigarettes. Int J Environ Res Publ Health.

[37] Jensen RP, Luo W, Pankow JF, Strongin RM, Peyton DH (2015). Hidden formaldehyde in E-cigarette aerosols. N Engl J Med.

[38] Kosmider L, Sobczak A, Fik M, Knysak J, Zaciera M, Kurek J, Goniewicz ML (2014). Carbonyl compounds in electronic cigarette vapours: effects of nicotine solvent and battery output voltage. Nicotine Tob Res.

